# Effects of topographical guidance cues on osteoblast cell migration

**DOI:** 10.1038/s41598-020-77103-0

**Published:** 2020-11-17

**Authors:** F. M. Refaaq, X. Chen, S. W. Pang

**Affiliations:** Department of Electrical Engineering, Centre for Biosystems, Neuroscience, and Nanotechnology, City University of Hong Kong, Kowloon, Hong Kong China

**Keywords:** Biomedical engineering, Biotechnology

## Abstract

Cell migration is a fundamental process that is crucial for many biological functions in the body such as immune responses and tissue regeneration. Dysregulation of this process is associated with cancer metastasis. In this study, polydimethylsiloxane platforms with various topographical features were engineered to explore the influence of guiding patterns on MC3T3-E1 osteoblast cell migration. Focusing on the guiding effects of grating patterns, variations such as etch depth, pattern discontinuity, and bending angles were investigated. In all experiments, MC3T3-E1 cells on patterned surfaces demonstrated a higher migration speed and alignment when compared to flat surfaces. The study revealed that an increase in etch depth from 150 nm to 4.5 μm enhanced cell alignment and elongation along the grating patterns. In the presence of discontinuous elements, cell migration speed was accelerated when compared to gratings of the same etch depth. These results indicated that cell directionality preference was influenced by a high level of pattern discontinuity. On patterns with bends, cells were more inclined to reverse on 45° bends, with 69% of cells reversing at least once, compared to 54% on 135° bends. These results are attributed to cell morphology and motility mechanisms that are associated with surface topography, where actin filament structures such as filopodia and lamellipodia are essential in sensing the surrounding environment and controlling cell displacement. Knowledge of geometric guidance cues could provide a better understanding on how cell migration is influenced by extracellular matrix topography in vivo.

## Introduction

Cell migration is a tightly regulated and essential process for normal development, wound healing, and tissue regeneration, as well as a key driver for the metastasis of cancer^1–4^. These biological processes are mediated by the extracellular matrix (ECM), an active component of living tissue that facilitates cell adhesion, cell to cell communication, and cell proliferation, to name a few^5,6^. Importantly, the ECM is known to influence cell migration track and speed through its topography and physical properties. During cancer development, cells have the ability to degrade the ECM and migrate away from the primary tumour, thus making cell migration a highly profound area of research^7^.

The guidance of cells through contact with their surroundings was found to be important as cells were observed to sense surface topographies at the microscale and subsequently, the nanoscale^8,9^. There is a plethora of evidence demonstrating the guidance of cells in two-dimensional (2D) microenvironments^10,11^. However, a growing number of studies have successfully demonstrated cell guidance within a three-dimensional (3D) microenvironment^12,13^. Studies using 3D platforms are on the rise as they closely mimic the ECM, therefore producing a more accurate and reliable representation of cell migration in vivo. Additionally, studies have manipulated feature dimensions such as width, etch depth, and spacing, as well as different patterns, as a means to identify the best form of topographical guidance. Other characteristics such as biochemicals and nano or micro scaled topographies, have also been shown to influence cell guidance^14,15^. It is long established that cells on flat surfaces have a tendency to move randomly and at a slower speed compared to patterned topographies^16,17^. Comparatively, gratings, the most commonly used topographical guiding pattern, have been shown to induce cell alignment in actin rich structures known as lamellipodia and filopodia^18^. Lamellipodia are large, sheet-like projections associated with cell displacement, whereas filipodia are spiky cytoplasmic projections which acts as a sensor and explores the microenvironment^19^. Various cellular structures including integrins are part of a larger complex known as focal adhesions (FAs) and also play a role in sensing the environment. These structures facilitate the interaction between the cytoskeleton and intracellular components within the ECM through a number of signalling pathways, ultimately resulting in changes in the cytoskeleton and subsequently, cell function^20,21^.

Given the vast range of topographies and features existed in living tissue, continuous topographies and structures may not accurate representations of the ECM as a whole. It is therefore important to investigate guiding patterns other than continuous gratings in order to fully understand cell migration. In this study, engineered platforms comprising of various surface topographies and altered feature characterisations were used to investigate the different guiding effects on MC3T3-E1 osteoblast cell migration. In this systematic study, cells were sensitive to small variations in topographical features, which in turn control their migratory behaviour. The effects of increasing etch depth on cell elongation and alignment were first investigated, followed by the influence of bends and pattern discontinuities on cell migration. Although patterned topographies on 2D platforms have been studied before, there is limited research on cell migration using the unique features shown in this study. The significant enhancement in cell migration speed and alignment shown in these results emphasises the importance of understanding the influence of these guiding patterns and topographic characteristics on cell migration. Thus, the findings of this study create an effective platform for the implementation of various topographic guiding patterns, which are shown to be successful in facilitating and controlling cell migration.

## Materials and methods

### Fabrication of engineered platforms

**Figure 1:**
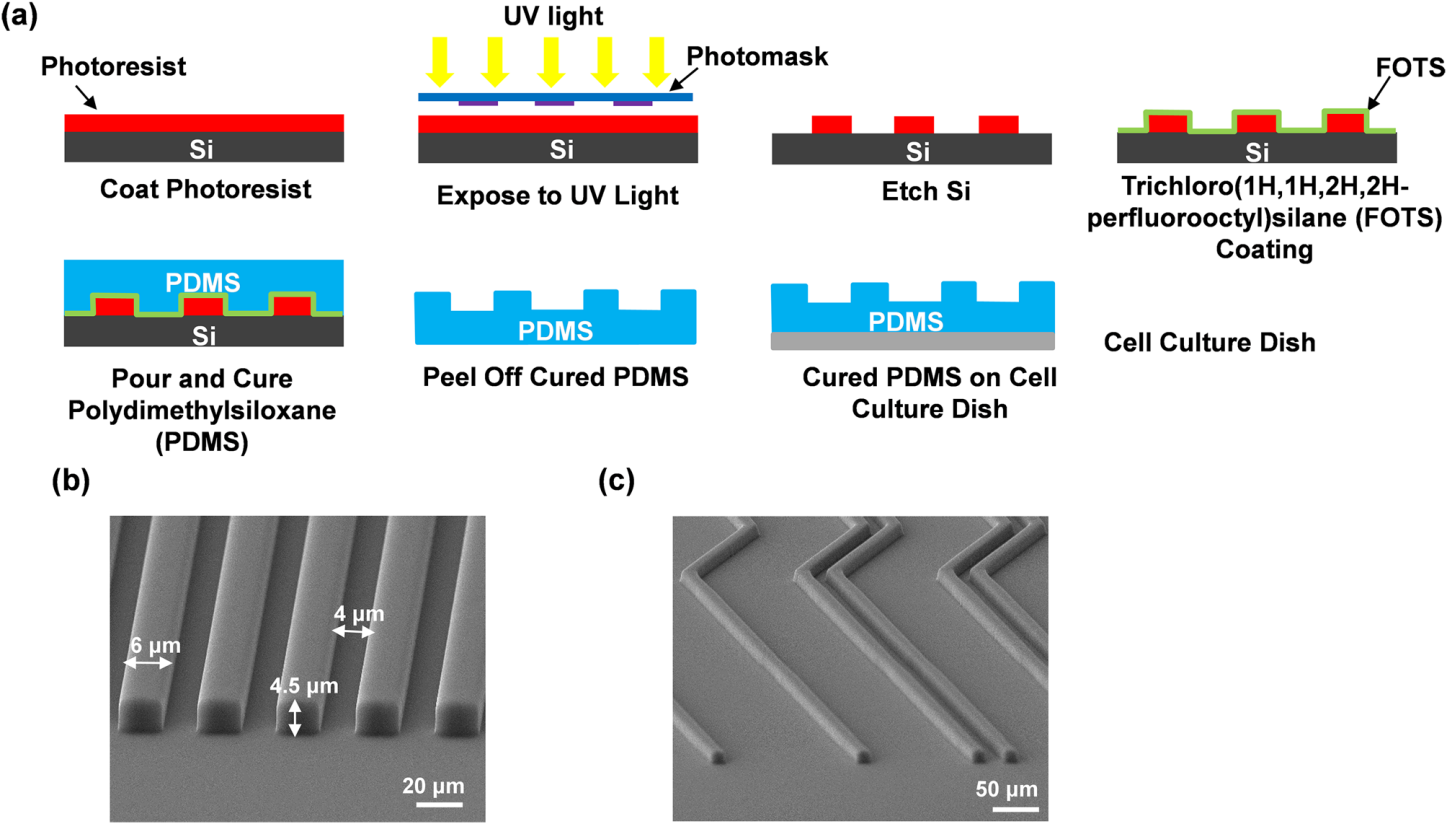
Fabrication of patterned PDMS platforms. (**a**) Patterns were transferred onto Si using photolithography and replicated onto PDMS by soft lithography. O_2_ plasma was used to bond PDMS to cell culture dish. Micrographs of (**b**) grating pattern with 6 μm ridges, 4 μm grooves, 4.5 μm depth and (**c**) bend density with 1 and 2 consecutive bends; 2 μm ridges and 4.5 μm depth.

Photolithography was used to transfer patterns onto a silicon (Si) wafer, followed by soft lithography to replicate the patterns onto a polydimethylsiloxane (PDMS) substrate as shown in Fig. 1a. Briefly, SPR6112B positive photoresist (Dow Corning) was spin-coated onto a Si wafer for 1 min at 3000 rotation per min and exposed to ultraviolet light for 6 s under a photomask. The Si wafer was immediately immersed in a developer solution for 25 s to remove the unexposed photoresist. The patterned Si was dried using N2 gas and baked on a hot plate at 120 °C for 10 min. The photoresist was then removed with acetone in an ultrasonic chamber before the etching process. To etch the 150 and 500 nm grating patterns, reactive ion etching (RIE) was applied using 21/2.5 sccm CF_4_/O_2_, 20 mTorr chamber pressure, and 150 W RF power for 6 min and 20 min, respectively. The 1 and 4.5 μm deep patterns were etched using a deep reactive ion etching (DRIE) system with 70/35 sccm C4F8/SF6, 10 mTorr chamber pressure, 600 W 13.56 MHz coil power and 10 W platen power for 2.0 min to etch 1 μm Si; and 138/11 sccm SF_6_/O_2_, 28 mTorr chamber pressure, 600 W 13.56 MHz coil power and 14.8 W platen power for 1.2 min to etch 4.5 μm Si. The PDMS platform was made by thoroughly mixing PDMS prepolymer and curing agent (Dow Corning Sylgard 184 Kit) at a 10:1 ratio before degassing at 20 °C for 15 min inside an ultrasonic chamber and then in a vacuum chamber for 30 min. The Si mold was coated with trichloro(1H,1H,2H,2H-perfluorooctyl)silane (FOTS) and baked on a hot plate at 80 °C for 30 min to facilitate easy demoulding of PDMS. PDMS was poured onto the Si mold and baked on a hot plate for 4 h at 80 °C for curing. The PDMS platforms containing the desired patterns were placed on a cell culture dish for time lapse imaging.

### Cell culture and immunofluorescence

MC3T3-E1 mouse osteoblast cells were maintained in Dulbecco’s modified Eagle medium (DMEM, Gibco 1X) supplemented with 10% fetal bovine serum, 1% penicillin–streptomycin and 1% GlutaMAX. The cells were kept in a 5% CO_2_ incubator at 37 °C and the medium was changed every 2–3 days. To stain the cells for fluorescent imaging, MC3T3-E1 cells on PDMS platforms were washed with 1% phosphate buffered saline (PBS) twice and fixed with 4% paraformaldehyde for 15 min at room temperature. Throughout the staining process, PBS was used to wash the cells in between each step. Cells were permeabilized in 0.1% Triton X-100 solution for 12 min before immersed in blocking solution with 1% bovine serum albumin for 30 min. Cells were then incubated with the primary antibody mouse anti-vinculin (Sigma-Aldrich) over night before being washed and incubated with secondary antibody Alexa Fluor 488 goat anti-mouse (Sigma-Aldrich) for 2 h. Following this, 4′,6-diamidino-2-phenylindole (DAPI, Sigma-Aldrich) was used as a counterstain to stain the cell nuclei for 30 min. After washing the cells in Alexa Fluor 555 phalloidin (Sigma-Aldrich) for 2 h to stain F-actin, the cells were subsequently washed with PBS thrice in preparation for imaging using a Nikon upright microscope.

### Time-lapse imaging

A 35 mm glass bottom confocal dish (SDL life Sciences) was treated with an O_2_ plasma for 1.5 min using a flow rate of 400 sccm O_2_, 400 sccm N2, 0.2 mbar pressure, and 200 W RF power with the purpose of cleaning the dish and making it hydrophilic. The PDMS platforms were then bonded onto the dish and treated under the same conditions in order to render the PDMS surface hydrophilic and create a suitable environment for cell growth. The platforms were immediately stored in deionized water to maintain hydrophilicity. 70% ethanol was used to wash the sample twice followed by 1% PBS. MC3T3-E1 cells maintained in DMEM were seeded onto the platforms for initial attachment at a density of 2.5 × 104 for 6 h. Prior to imaging, the medium was changed to a CO_2_ independent medium. Cells were maintained at 37 °C for the duration of the imaging and observed on the platforms every 5 min for a period of 16 h using a Nikon upright microscope.

### Scanning electron microscopy

After MC3T3-E1 cells were seeded onto the patterned PDMS platforms for approximately 24 h, the cells were fixed with 4% PFA for 15 min at room temperature and underwent alcohol dehydration using increasing concentrations of ethanol (30%, 50%, 70%, 80%, 90%, 95%, and 100%). The samples were dried using the critical point dryer and coated with a thin layer of Au before being imaged using a scanning electron microscope (Hitachi SU5000).

### Data analysis

MC3T3-E1 cells were tracked using Image J software (version 1.48v) with the manual tracking plugin. Microsoft Excel and GraphPad Prism (version 8.4.3) were used to perform statistical analysis using one-way analysis of variance (ANOVA) and Tukey’s post hoc test (p < 0.05). All results presented are mean ± standard error of mean (SEM). Tracked cells consisted of single live cells that did not multiply or interact with another cell. Cells that died were also excluded from analysis. To measure aspect ratio, the freehand selection tool was used to trace around the perimeter of the cell. Parameters such as area, perimeter, shape descriptors, and fit ellipse were selected in order to calculate aspect ratio. Cell aspect ratio was defined as the major axis divided by the minor axis. Directionality ratio was calculated by the straight-line distance between the starting and ending points of trajectory divided by the total distance that the cell actually travelled. Root mean square (RMS) of distance was calculated as the average displacement of cells over the 16 h time-lapse period^22^. Each experiment was repeated a minimum of three times to maintain accuracy and produce reliable results.

## Results and discussion

### Topographical guiding patterns

Osteoblast cell migration has been the primary focus of many topographical guiding effect studies^13,23–25^. In this paper, three aspects of surface topography were used to investigate the impact of different guiding patterns on MC3T3-E1 osteoblast cell migration. Various PDMS platforms including “Gratings” with 6 μm ridges, 4 μm grooves, and different etch depths as shown in Fig. 1b; discontinuous patterns, namely “Ridges”, “Ridges & Pillars” and “Oblongs”; and bent gratings, “135° Bends”, “45° Bends”, and “Bend Density” as shown in Fig. 1c, were designed and fabricated. Discontinuous patterns had an etch depth of 1 μm and the level of discontinuity varied between the patterns. “Ridges” are broken up gratings, 50 μm long and a 5 μm gap between each ridge. “Ridges & Pillars” have 2 rows of pillars in between each ridge and subsequently have the most pattern disruption. “Oblongs” are shorter ridges with a length of 10 μm and 5 μm width, and is therefore anisotropic. Patterns with different bending angles had an etch depth of 4.5 μm. While comparing the two different bending angles, the effect of an increased grating density with a bending angle of 135° was also studied. In all three experimental groups, MC3T3-E1 cells were also compared to those seeded on a flat surface and on gratings with the same etch depth as a control.

### Gratings with different etch depth

#### Migration speed and directionality

**Figure 2:**
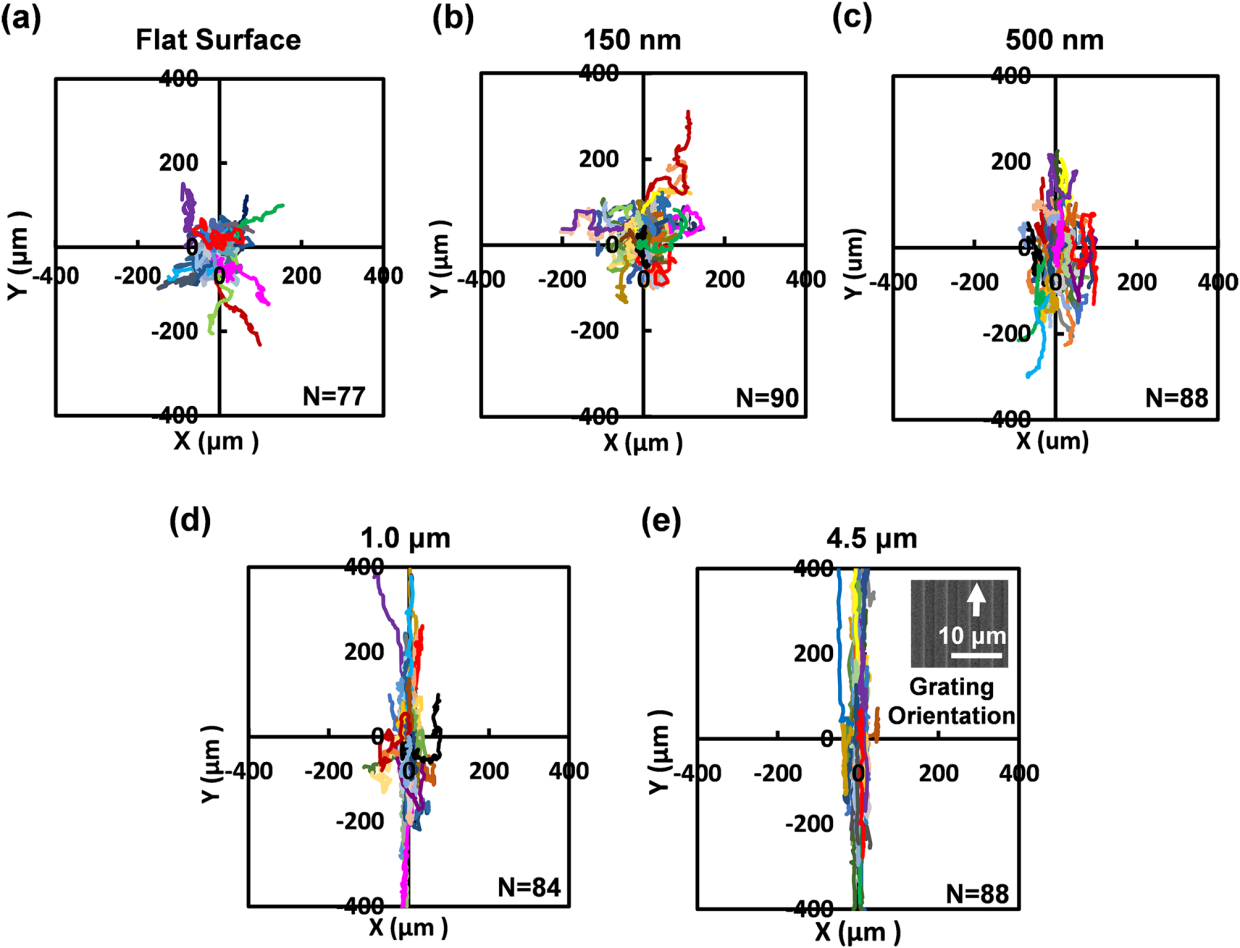
Cell migration trajectories of MC3T3-E1 cells showing the guiding effect of gratings with different groove depths on (**a**) flat surface, (**b**) 150 nm, (**c**) 500 nm, (**d**) 1.0 μm, and (**e**) 4.5 μm.

In this paper, a systematic approach was implemented to investigate the effects of increasing etch depth on grating patterns with the same dimensions. While the guidance of cells along grating patterns have been shown in the past, a clear effect of grating depth varying from less than 1 μm to a few μm has not been studied^11,16,26–29^. Using both RIE and DRIE technologies, PDMS platforms were fabricated with different depths of 150 nm, 500 nm, 1 μm, and 4.5 μm. Cells on the flat surface were also observed as a control. As shown in Fig. 2, MC3T3-E1 cells became better guided along the grating orientation as the grating depth increased from 150 nm to 4.5 μm. On the 150 nm shallow gratings, cell trajectories appear to be random and similar to the random trajectories shown on the flat surfaces. The first signs of cell guidance are shown when the depth of gratings was increased to 500 nm, signifying that the trajectories were influenced by the change in grating depth. Furthermore, there is a significant increase in cell guidance as the grating depth doubled to 1 μm and increased further to 4.5 μm. It is therefore evident that the gradual escalation in the guidance of MC3T3-E1 cells by the grating patterns is due to the increase in etch depth. It is plausible that the sidewalls on deeper etched patterns provide more surface area for contact guidance, hence enabling more FAs to be formed along the grating and the capability for cells to be guided better. Though there is a small increase in the guidance of cells between the 1 and 4.5 μm deep patterns, there appears to be a saturated effect of depth dependence, where directionality will presumably remain consistent when the etch depth is at least 1 μm. This study clearly demonstrates the distinguishable progression of depth associated guidance in the cell migration trajectories using grating depths ranging from a shallow etch of 150 nm to a deeper etch of more than 1 μm.

**Figure 3:**
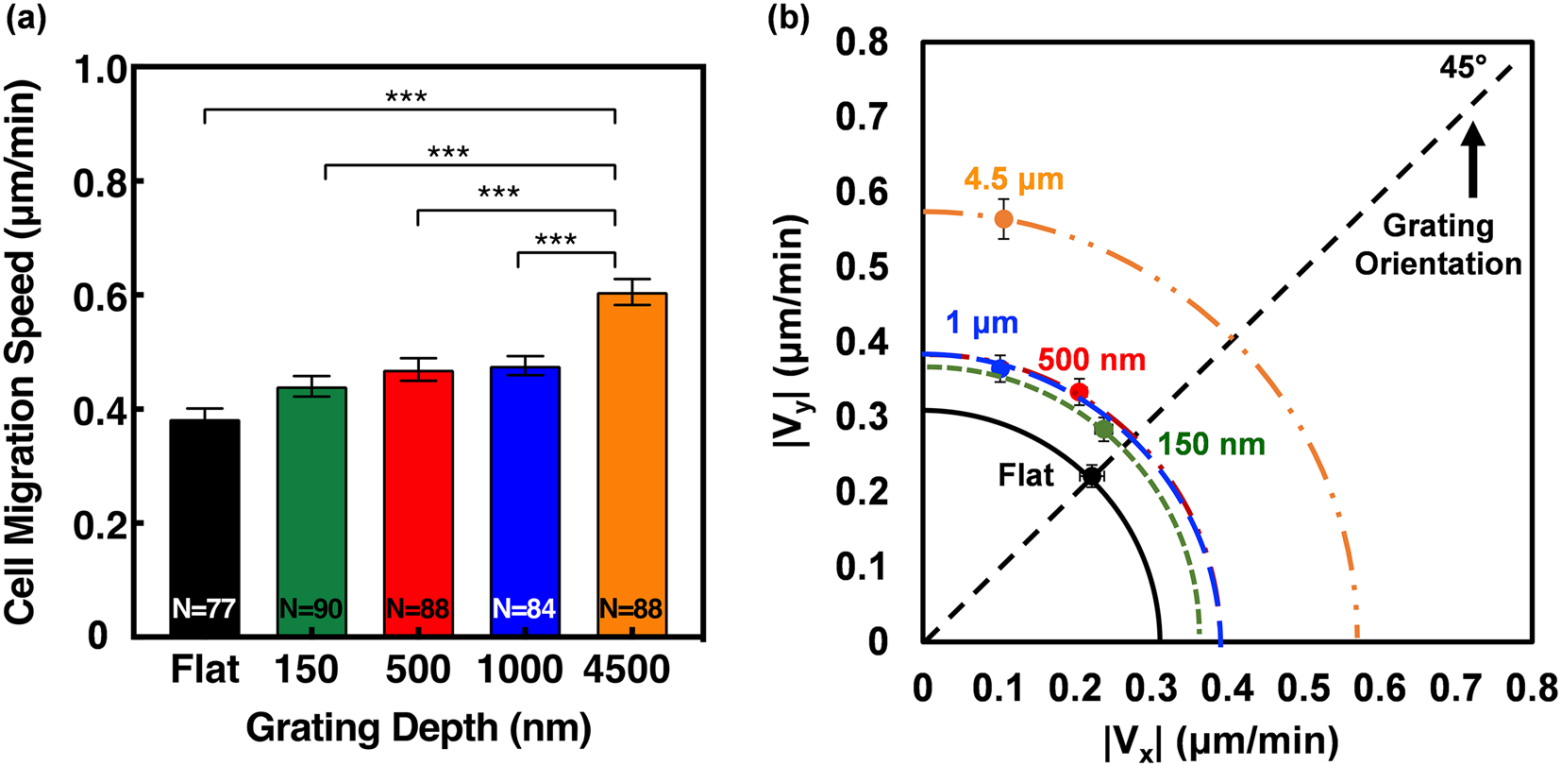
(**a**) Cell migration speed of gratings with different depths. (**b**) Cell migration speed of gratings in x and y directions with different groove depths. All error bars are mean ± SEM. One-way ANOVA and Tukey’s post hoc test were performed to test for statistical significance (***p < 0.001).

Interestingly, Fig. 3a shows that the cell migration speed difference between gratings with depths of 150 nm, 500 nm, and 1 μm were statistically insignificant despite the clear differences in trajectories. However, migration speed on the 4.5 μm deep gratings was significantly higher than the shallower depths, with a speed of 0.61 μm/min compared to 0.48, 0.47, and 0.44 μm/min for 1 μm, 500 nm, and 150 nm etch depths. In order to understand the directional preference of each grating pattern, cell migration speed in the x and y directions were also looked at, as shown in Fig. 3b. The alignment angle, θ, was measured as an indicator of cell directionality, where a smaller angle represents directional migration along the grating orientation and larger angles represent a less directional migration. MC3T3-E1 cells on flat surfaces typically have an alignment angle of 45°, which is an indicator of randomness. On gratings with 4.5 and 1 μm depth, the angles of alignment were 11° and 17°, respectively. As the etch depth became shallower, the angle of alignment increased towards randomness, with cells on the 150 nm shallow gratings exhibiting an alignment angle of 41°. Similar to the cell migration trajectories, cells on the gratings with 150 nm etch depth were the least directional and comparable to the random migration shown on the flat surfaces. While there was a strong cell directionality induced by deeper etched gratings, cell migration speed remained relatively constant until the etch depth became 4.5 μm, suggesting that migration speed and directionality are not significantly correlated. Other studies have demonstrated limited changes in cell migration in grating patterns with an etch depth less than 2 μm^16,26,28,30^. The results of this study are therefore crucial in demonstrating the definite relationship between cell alignment and guidance with increasing etch depths, irrespective of cell migration speed.

#### Grating depth affects cell elongation

**Figure 4:**
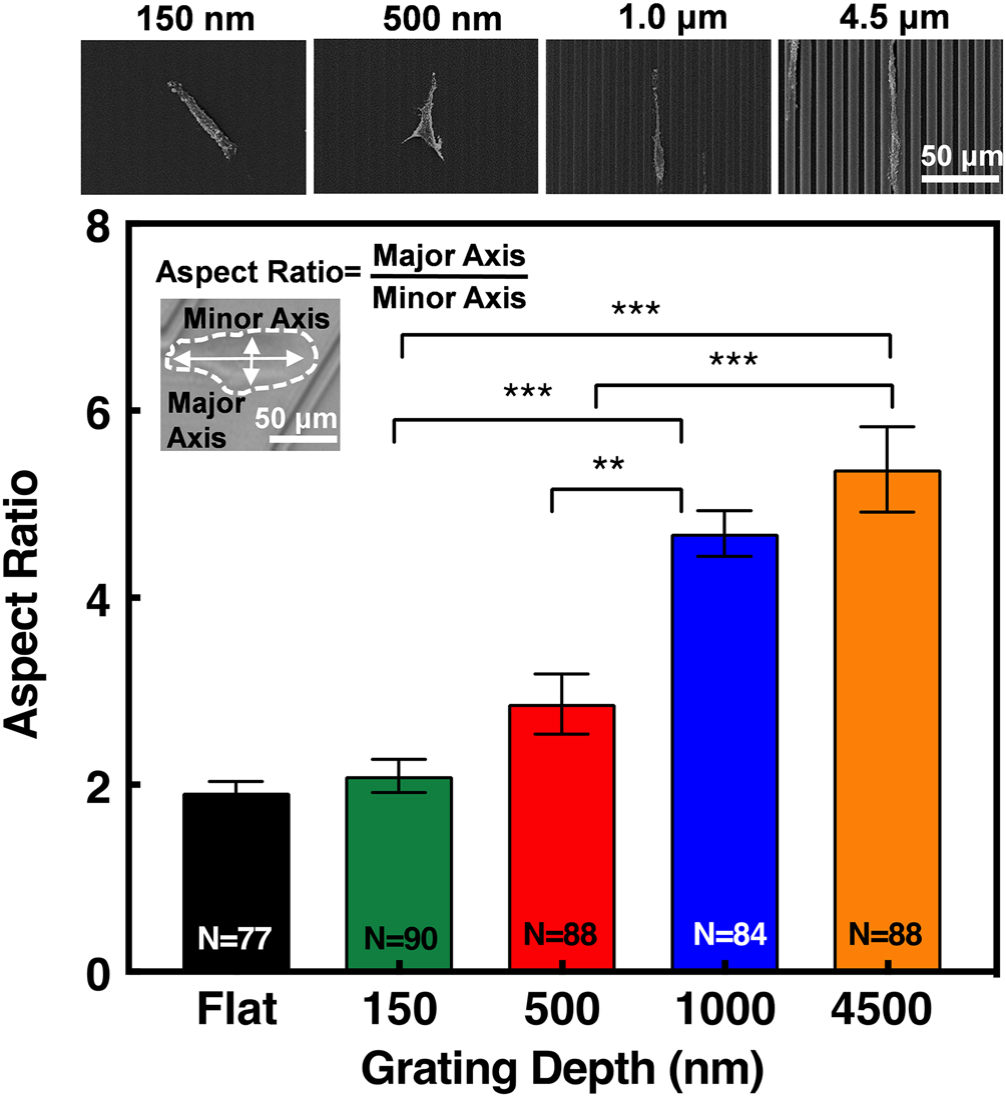
Aspect ratio of MC3T3-E1 cells on gratings with different depths. Cell shape was observed at 0, 4, 8, 12, and 16 h and the mean aspect ratio was calculated. One-way ANOVA and Tukey’s post hoc test with **p < 0.01 and ***p < 0.001. Micrographs represent MC3T3-E1 cell elongation on different gratings.

To get a better understanding of how etch depth influences the directionality and migration speed of MC3T3-E1 cells, the aspect ratio of all tracked cells was calculated^27^. The shape of each cell was traced at 0, 4, 8, 12, and 16 h and averaged in order to obtain an accurate measurement of elongation over the 16 h time lapse. Typically, MC3T3-1 cells were quick to become elongated on the deeper etch depths. However, on flat surfaces and 150 nm shallow gratings, cell morphology was rounder and took longer to elongate. MC3T3-E1 cell protrusions also appeared to be spreading in all directions on the flat and shallow etched platforms, whilst cells on the deeper etch platforms showed leading and trailing protrusions along the grating direction. As shown in Fig. 4, the aspect ratio increased as the depth of the gratings increased, with MC3T3-E1 cells significantly more elongated on gratings with 4.5 and 1 μm depth compared to the shallower depths and flat surfaces. Cells had an aspect ratio of 5.4 on 4.5 μm deep platforms compared to 2.1 on 150 nm shallow platforms. On average, MC3T3-E1 cells on the 4.5 μm deep platforms were 2.4 times more elongated than cells on 150 nm, and 2.8 times more elongated than flat surfaces. The corresponding scanning electron micrographs reveal typical MC3T3-E1 cells aligning along the deeper etched gratings, further demonstrating how cells became more elongated and guided as the etch depth increased.

The basic phenomenon of contact guidance is shown here where directed cell migration is a result of protrusions from the cell membrane^19^. According to the theory of mechano-transduction, actin filaments of the cytoskeleton transfers information to the cell nucleus, resulting in the repositioning of the chromosomal interphase centromere^31–35^. Given this, the correlation of cell elongation and alignment with increasing etch depth may occur because the larger etch depth provided more surface area for cell membrane protrusions to make contacts with. These interactions could induce the morphological changes in osteoblast cell cytoskeleton as observed on deeper etched patterns. Hence, cells are more elongated and oriented on deeper etched patterns as shown in Fig. 4. It is evident that cell elongation is a function of etch depth, therefore these results produce a better understanding of cell migration by further emphasising the visibly imperative correlation between cell elongation and alignment with increasing etch depths.

### Patterns with different discontinuities

**Figure 5:**
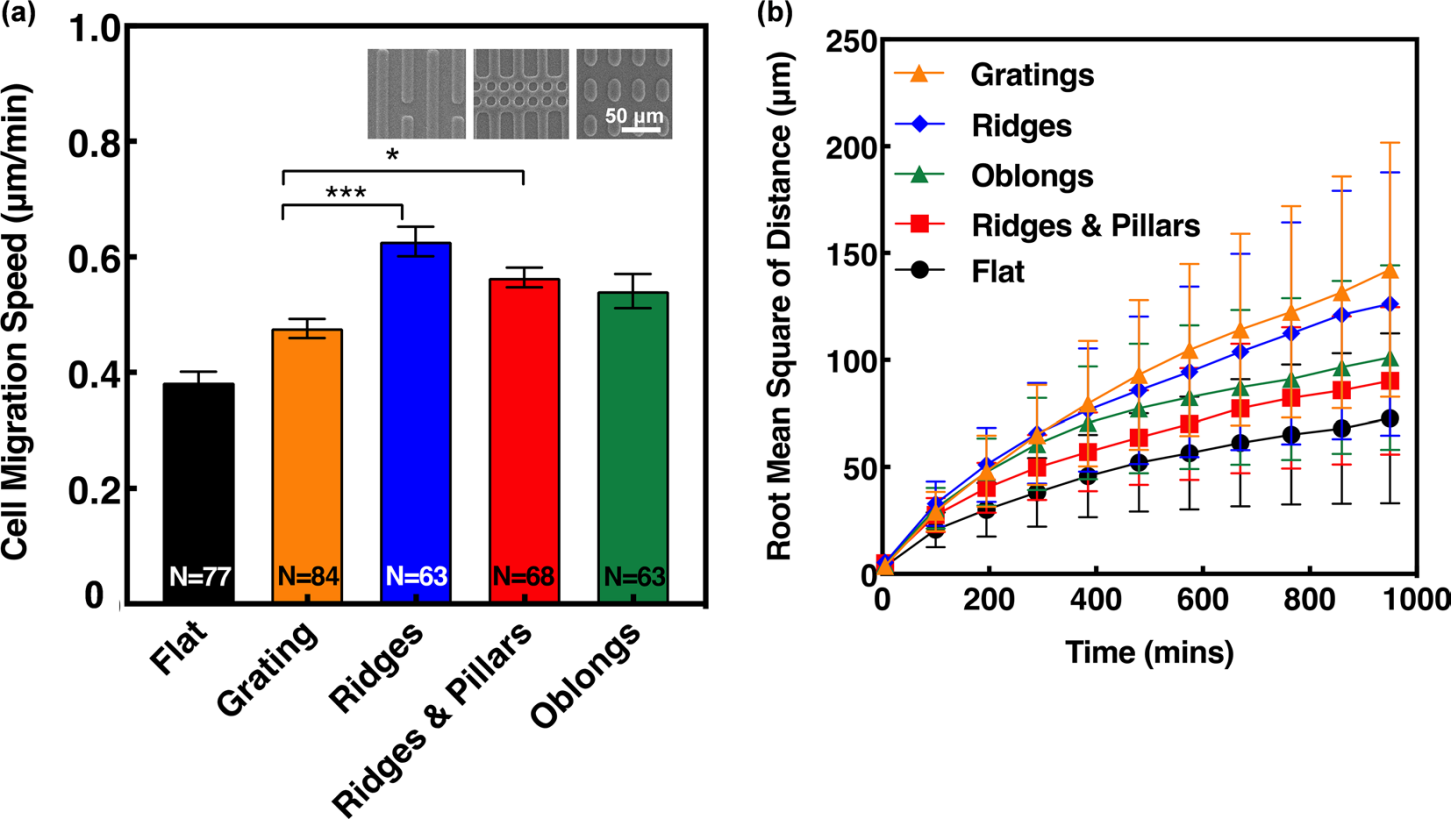
(**a**) Migration speed of MC3T3-E1 cells on patterns with different levels of discontinuity. Micrographs present different patterns with ridges of 4 μm ridges, 5 μm spacing; ridges & pillars of 6 μm ridges, 5 μm spacing; and oblongs of 5 μm width, 10 μm length, 5 μm spacing. The depth of all patterns is 1 μm (One-way ANOVA and Tukey’s post hoc test with *p < 0.05 and ***p < 0.001). (**b**) RMS of displacement distance for MC3T3-E1 cells on discontinuous guiding patterns.

The level of pattern discontinuity was investigated and shown to influence migration speed and directionality of MC3T3-E1 cells. Overall, cell migration speed on the discontinuous patterns was higher when compared to gratings, with ridges, and ridges & pillars significantly higher, as shown in Fig. 5a. The organisation of the ridges pattern produces a continuous grating effect, which presumably should induce a similar migration speed as found on the gratings. However, the higher speed of 0.63 μm/min on the ridges pattern compared to 0.48 μm/min on the grating pattern could be attributed to the discontinuous gaps found between the ridges, giving MC3T3-E1 cells breaks in continuous contacts and more opportunities for migration. The filopodia have the ability to sense across these 5 μm gaps and migrate onto parallel ridges, as well as having the choice to continue the grating path ahead, which could explain the higher migration speed found on the ridges. On the ridges & pillars, migration speed was 0.56 μm/min compared to 0.48 μm/min on gratings. Though ridges & pillars are a highly disrupted pattern, the higher migration speed suggests that extension of filopodia may use the pillars as a form of contact guidance, rather than it being an obstruction. In terms of the oblongs, the 5 μm spacing between the patterns appears to be sufficient for MC3T3-E1 cells to extend their filopodia and form FAs, thus influencing cell migration.

**Figure 6:**
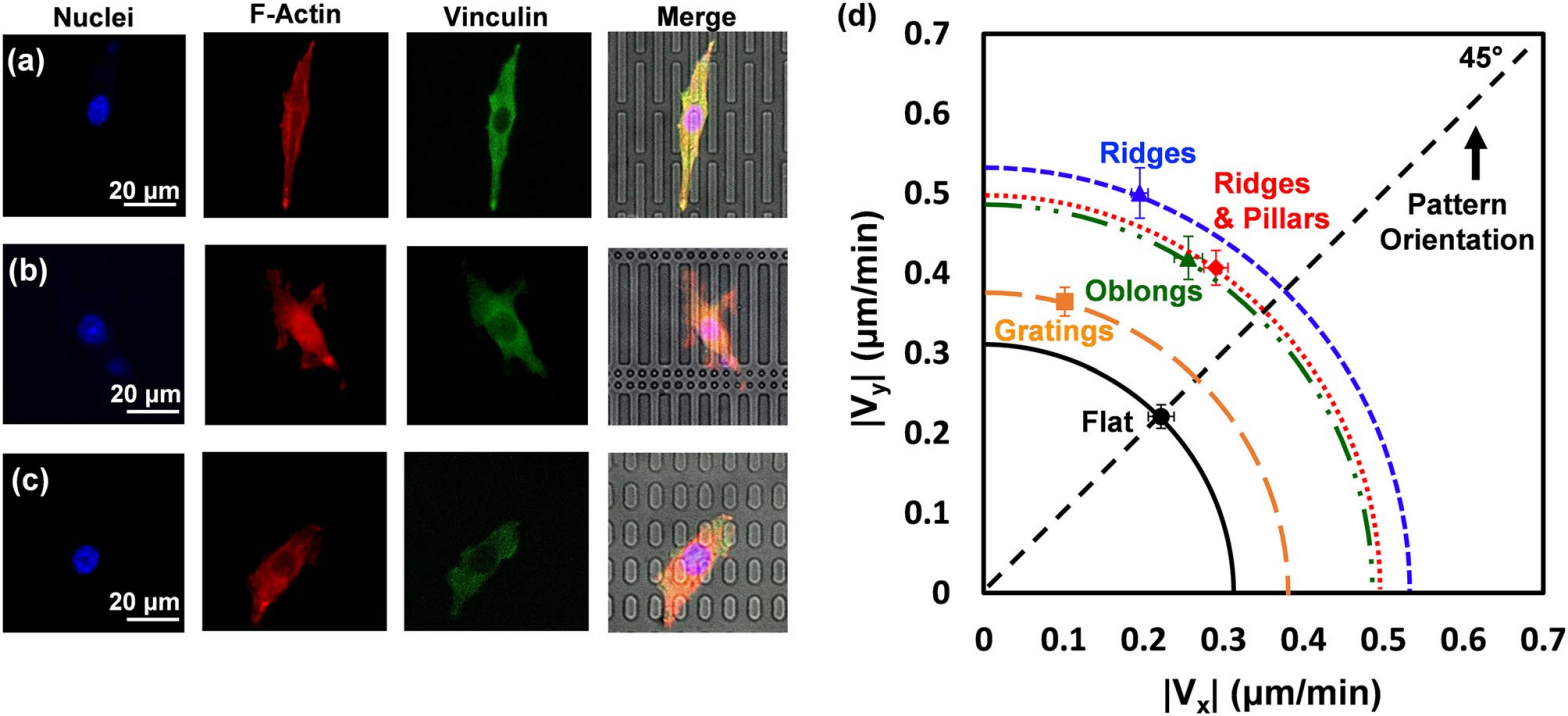
Cells were stained to observe nuclei, actin filaments, and cytoskeleton under fluorescent imaging with cells on (**a**) ridges, (**b**) ridges & pillars, and (**c**) oblongs. (**d**) Comparison of MC3T3-E1 cell migration speed in x and y directions on discontinuous guiding patterns.

RMS of distance showed the displacement of M3T3-E1 cells on each guiding pattern over the 16 h time lapse as shown in Fig. 5b. On the gratings pattern, cell displacement increased from 3.9 to 142 μm in 965 min compared to an increase from 5.3 to 126 μm in ridges. There is a small difference in cell displacement between ridges and gratings, yet significant differences in cell migration speed. This deviation in results is likely due to the directionality preferences of the cell movement on these patterns as RMS of distance is also an indicator of directionality. RMS of distance is calculated using the change in cell displacement from one point to another point in 5 min intervals, independent of any diversions in migration direction. As will be shown later in Fig. 6, cells on gratings had a slightly higher directionality preference compared to those on the ridges, which occurred when cells had the opportunity to migrate between parallel ridges, thus influencing the cell migration directionality. Furthermore, the smaller RMS of distance as shown by the ridges & pillars is likely due to the higher level of disruption within the pattern, making it difficult for cells to migrate further than cells on the other patterns. Taking this into consideration, RMS of distance coupled with directionality and speed are crucial in showing the disparities in cell displacements between the discontinuous patterns. The results in this study suggest that when an increase in speed is associated with a decrease in RMS of distance, it is due to the directional changes that occurs in discontinuous patterns. On the contrary, larger displacement as shown on continuous grating indicates a higher level of directionality.

Figure 6 shows the fluorescent and bright field images of stained MC3T3-E1 cells on (a) ridges, (b) ridges & pillars, and (c) oblongs. MC3T3-E1 cells were stained in order to visualise the actin cytoskeleton organisation as well as the FAs behaviour on each discontinuous pattern. Cells on the ridges pattern were more elongated with actin fibres aligned along the axis, in comparison to the other two discontinuous patterns. As shown on in Fig. 6a, the MC3T3-E1 cell is more polarised on the ridges as there are clear leading and trailing ends of the cell, which drive membrane protrusion. The leading end is shown to be turning towards parallel ridges, demonstrating the influence of a gap between patterns on filopodia extension as discussed earlier. From the fluorescent images of ridges & pillars and oblongs shown in Fig. 6b and c, cells are less polarised, and the organisations of cellular components are less asymmetric than the ridges. This complements the directionality preferences of the different patterns shown in Fig. 6d, where ridges were the most oriented discontinuous pattern with a directional angle of 21°, followed by oblongs and ridges & pillars with angles of 32° and 36°, respectively. The spacing between oblongs allowed cells to extend in both the x and y directions, whereas the ridges and gratings heavily influenced migration in the y direction. Furthermore, the high level of disruption in the ridges & pillars pattern impacted the ability of MC3T3-E1 cells to migrate easily in the y direction, as shown in Fig. 6d. This study explores different levels of pattern discontinuity on cell migration, which is related to how filopodia are probing and FAs are tuned to topographical intervals^18^. As the spacing between patterned discontinuities was 5 μm or less, filopodia extension was able to contact the discontinuous pattern features and influence migration. The findings of this study provide crucial insight into how mimicking ECM using different discontinuous patterns can have significant impacts on cell migration speed and direction.

### Gratings with bends

#### Bending angle effect

**Figure 7:**
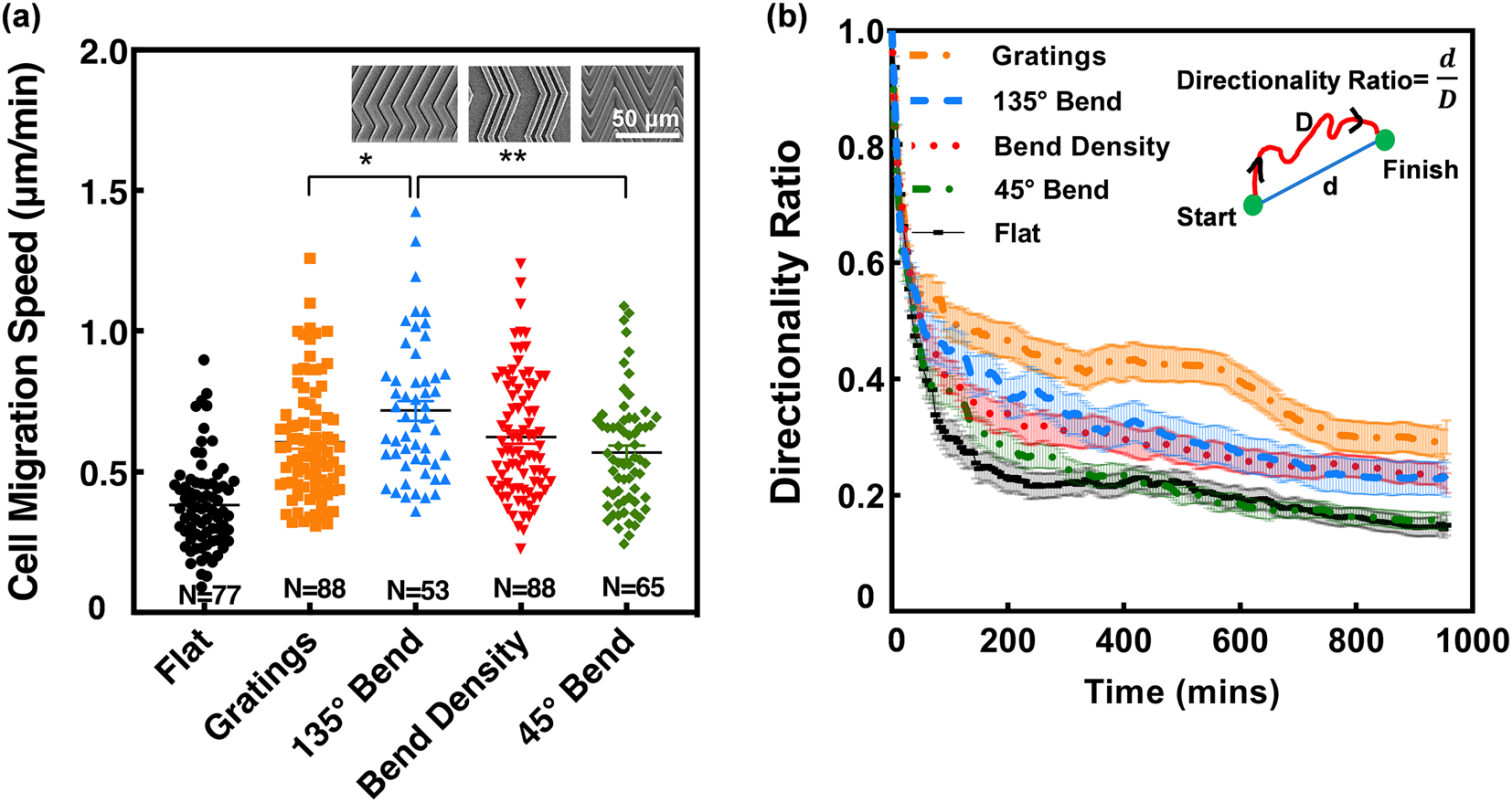
(**a**) Cell migration speed of MC3T3-E1 cells on bent guiding patterns with bent angles of 135° and 45°. Micrographs present different bent patterns of gratings of 6 μm ridges and 4 μm grooves; 135° bend of 4 μm ridges, 1 μm grooves, 100 μm length and 25 μm bend length; bend density of 2 μm ridges, 3 μm grooves, 200 μm length, and 50 μm bend length; and 45° bend of 4 μm ridges, 1 μm grooves, 100 μm length, and 75 μm bend length. The depth of all patterns is 4.5 μm. One-way ANOVA and Tukey’s post hoc test with *p < 0.05 and **p < 0.01. (**b**) Directionality ratio of patterns with bends over 16 h time-lapse.

In this study, patterns with different bending angles were found to influence the migration of MC3T3-E1 cells. Overall, migration speed was higher on patterns with obtuse bends as shown in Fig. 7a. The mean cell migration speed on 135° bends was 0.72 μm/min, which was significantly higher than 45° bends of 0.57 μm/min as well as gratings of 0.61 μm/min, with the same etch depth, whereas migration speed on bend density was 0.62 μm/min. While bend density also had a bending angle of 135°, the slower migration speed is likely due to the large spacing between the bent gratings. These findings demonstrate that migration speed was enhanced by bent gratings with an obtuse angle, irrespective of spacing between the patterns, compared to an acute angle, suggesting that MC3T3-E1 osteoblast cells are sensitive to bends. On 135° bends, there is a larger surface area for cells to form contact with, which facilitated cells to move forward and past the bends. As a result, 135° bends were more directional and induced a higher cell migration speed. However, on 45° bends, there is a smaller surface area and less contact points for cells to be effectively guided. The sharp bend prevented cells from moving forward, thus creates a trapping effect and the inclination to change direction.

Additional contributing factors to the overall enhancement of migration speed include the effect of feature dimensions such as width and depth. Feature dimensions are known to influence migration, although, the specific influence depends on the cell line, topographical pattern, and feature size. Studies have proposed that smaller groove widths guide cells better than grooves with larger widths^20^. Coupled with the results on the influence of cell migration with increasing etch depth demonstrated in this study, it is possible that the dimensions of the bent gratings with 4.5 μm depth, 1 μm groove width, and 135° bends simultaneously enhanced cell migration.

During the time lapse imaging, the MC3T3-E1 cells showed that cells were better guided by the 135° bent gratings compared to 45° bends. In Fig. 7b, directionality ratio was calculated as an indicator of cell persistence over the 16 h time lapse. A directionality ratio of 1 means cells migrated in a straight line. The 45° bends induced a faster decay in directionality as cells were inclined to reverse more on acute bends compared to the 135° obtuse bends, which is similar to a previous study^10^. While gratings had the highest directionality ratio, followed by patterns with 135° bends, the directionality ratio on 45° bends was similar to a flat surface despite the large differences in migration speed. The present findings suggest that cell reversals influence directionality ratio as MC3T3-E1 cells were shown to reverse more on 45° bends compared to 135° bends. On 135° bends, cells reversals were less than 45° bends, and therefore had a higher directionality ratio. However, the deviation between gratings and 135° bends likely occurred due to the presence of bend induced reversals, thus causing gratings to have a higher directionality ratio. As mentioned earlier, cell migration directionality does not have a correlation with migration speed.

Furthermore, the impact of grating density and spacing between the bent gratings were also investigated. Cell migration speed for the 135° bent gratings with different consecutive bends was similar despite the increased spacing between bent gratings as shown in Supplementary Fig. S1. While MC3T3-E1 cells have a cell body size between 20–50 μm and the gap between the consecutive bend densities ranges from 30 to 42 μm, it is possible that the filipodia were able to extend and contact the adjacent groups of bent gratings to move, without affecting the overall cell migration speed^36^.

#### Number of reversals

**Figure 8:**
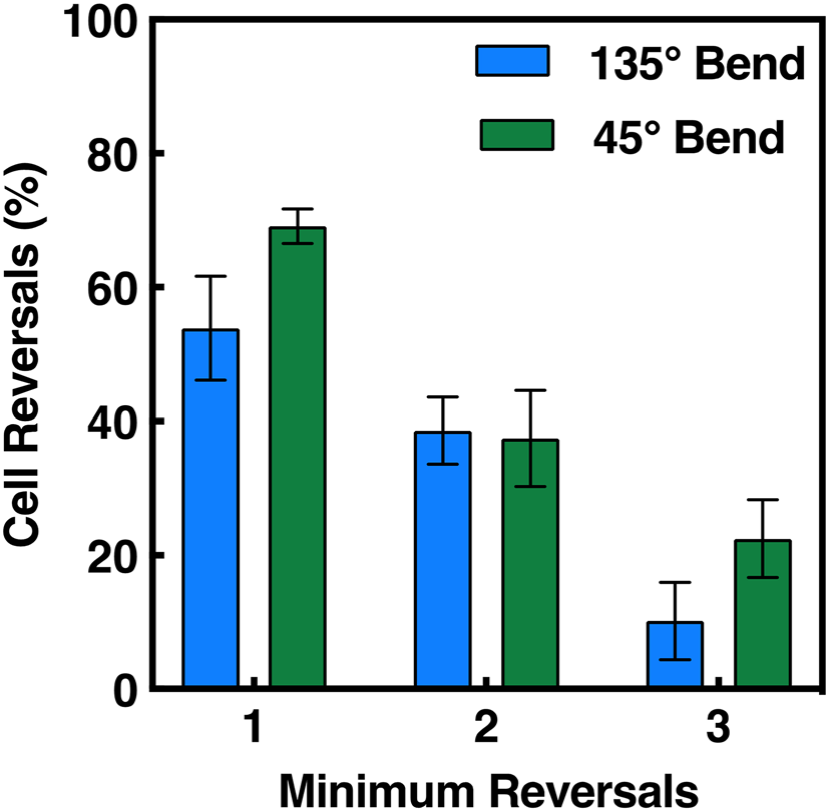
Minimum percentage of cell reversals of MC3T3-E1 cells on patterns with 45° bends (N = 65) and 135° bends (N = 53). Cell reversals were calculated by number of cells that reversed by 180° divided by total number of cells on patterned platforms. 2-way ANOVA and Sidak’s post hoc test indicated no significant differences between number of reversals and bending angle (p > 0.05).

During the 16 h time lapse, the number of cell reversals on patterns with 135° and 45° bends was monitored and the percentages are shown in Fig. 8. Cell reversals is defined as a cell that reversed the migration directionality 180° at a bend. The findings revealed that cells on 45° bends were more inclined to reverse migration direction. In fact, there were 105 cell reversals on 45° bends, with one cell reversing as many as 9 times, compared to just 60 cell reversals on 135° bends. 69% of MC3T3-E1 cells reversed at least once on 45° bends compared to 54% on 135° bends. Additionally, cells on 45° bends were 10 times more likely to migrate onto adjacent bent gratings compared to 135° bends. Occasionally, as cells approached the bent gratings on patterns with 45° bends, they reversed and migrated to the second bend at the end of the pattern, where they reversed at least one more time. 22% of cells reversed at least three times on 45° bends compared to 10% on 135° bends, emphasizing the high level of reversing capabilities that MC3T3-E1 cells may experience on acute bends. This suggests that a bending angle of 45° may be too sharp for cells to continue migrating along the same direction, thus enabling cell reversals. Despite the differences in reversal behaviour, it is evident in the present findings that bends in gratings induces the opportunity of reversals in MC3T3-E1 cells. This further emphasizes the sensitive nature of cells interacting with the surrounding microenvironment and the effect of bends on cell migration reversals.

## Conclusion

In the present study, the influence of different surface topographies on the migration of MC3T3-E1 osteoblast cells were investigated. 2D platforms were achieved by microfabrication technology using lithography, dry etching, and PDMS replication, which proved to be a simple and efficient method to explore cell migration on a large scale using a variety of topographies. The patterned platforms had three focus points including etch depth, pattern discontinuity, and bending angles. The effect of increasing etch depth led to a higher degree of cell alignment and elongation, whereas discontinuous elements and bending angles influenced migration speed, directionality, and cell reversals. The increase in surface area of gratings with deeper etch depths enabled membrane protrusions to form more contact guidance and enhance directional migration. These protrusions were vital in exploring the surrounding microenvironment and demonstrated the increased sensitivity to discontinuous patterns. Patterns with 45° bends induced more cell reversals compared to 135° bends, emphasizing the reversal inducing characteristic of acute bends. The findings in this study exhibit a novel understanding on cell migration on engineered platforms and highlight the impact of small variations in guidance cues on osteoblast cells, thus providing a valuable tool for the progression of controlling cell movement and separation in future research.

## Supplementary information

Supplementary Figure S1.
