# Supplementary material for: Effects of topographical guidance cues on osteoblast cell migration
Source: Sci Rep. 2020 Nov 17;10:20003. doi: 10.1038/s41598-020-77103-0 (PMC7672072; doi:10.1038/s41598-020-77103-0)
Supplement: Supplementary file 1 — Supplementary Figure S1. [file 41598_2020_77103_MOESM1_ESM.pdf]

Effects of Topographical Guidance Cues on Osteoblast Cell Migration

F. M. Refaaq, X. Chen, and S. W. Pang*

Department of Electrical Engineering

Centre for Biosystems, Neuroscience, and Nanotechnology

City University of Hong Kong, Kowloon, Hong Kong, China

*Corresponding Author:

S. W. Pang (pang@cityu.edu.hk)

Department of Electrical Engineering

City University of Hong Kong

Kowloon, Hong Kong, China

Phone: +852 3442 9853

Fax: +852 3442 0562

Supplementary Materials

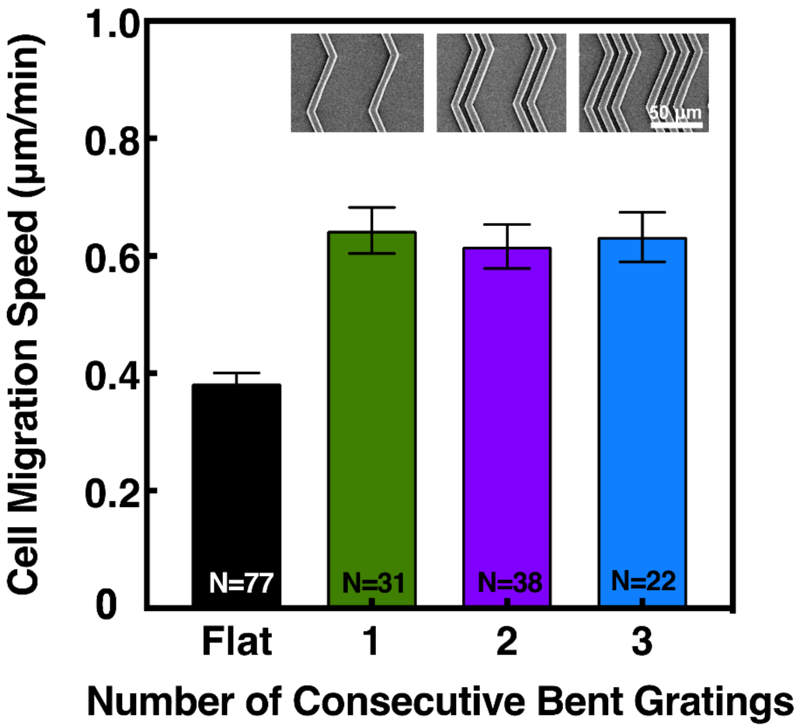

Supplementary Fig. S1. Speed of MC3T3-E1 cells on bent gratings with increasing bent density. Spacing between 1 consecutive bent grating is 42 µm; spacing between 2 consecutive bent gratings is 36 µm; and spacing between 3 consecutive bent gratings is 30 µm. One-way ANOVA and Tukey's post hoc test indicated no significant differences between bend density groups ($p>0.05$).
